# Metformin Attenuates Myocardial Ischemia–Reperfusion Injury in Rats by Modulating JNK Pathway and Inhibiting PANoptosis Mechanisms

**DOI:** 10.1155/cdr/4642838

**Published:** 2025-10-06

**Authors:** Biao Hou, Xuejian Hou, Liyue Zhang, Tingting Liu, Yang Li, Ran Dong

**Affiliations:** ^1^Coronary Artery Disease Surgical Center, Capital Medical University Affiliated Anzhen Hospital, Beijing, China; ^2^Capital Medical University, Beijing, China; ^3^Cardiac Surgery Center, Capital Medical University Xuanwu Hospital, Beijing, China; ^4^The Key Laboratory of Remodeling Cardiovascular Diseases, Ministry of Education, Beijing, China; ^5^Collaborative Innovation Center for Cardiovascular Disorders, Beijing, China; ^6^Beijing Institute of Heart, Lung and Blood Vessel Diseases, Beijing, China

**Keywords:** JNK, metformin, myocardial ischemia–reperfusion injury, PANoptosis

## Abstract

**Purpose:**

Myocardial ischemia–reperfusion injury (MIRI) is a condition in which the heart is aggravated when blood flow is restored after tissue damage caused by ischemia. Metformin (Met), a widely prescribed antidiabetic medication, has demonstrated promising cardioprotective properties, particularly through its anti-inflammatory, antiapoptotic, and metabolic regulatory mechanisms. This study investigates the cardioprotective effects of Met in a rat model of MIRI, focusing on its modulation of the c-Jun N-terminal kinase (JNK) pathway and inhibition of PANoptosis mechanisms.

**Methods:**

The proportion of myocardial infarction was determined by triphenyl tetrazole chloride (TTC) staining. Wheat germ agglutinin (WGA) staining is used to determine whether myocardial cells are hypertrophic and other pathological conditions. Serum markers of myocardial injury along with inflammatory cytokines were measured by enzyme-linked immunosorbent assay (ELISA). Immunofluorescence and Western blotting were employed to evaluate myocardial cell PANoptosis and the involvement of the JNK pathway.

**Results:**

After MIRI, TTC staining revealed apparent myocardial infarction, and WGA staining showed significant myocardial cell hypertrophy. There was also a marked increase in myocardial injury markers, inflammatory factors, and reactive oxygen species (ROS). Met significantly reduced the myocardial injury area and notably lowered serum levels of c-TnI, CK-MB, LDH, IL-6, TNF-*α*, and ROS. Immunofluorescence and Western blot analyses demonstrated that Met attenuates myocardial cell PANoptosis by inhibiting JNK phosphorylation and reducing ROS generation. The cardioprotective effect of Met was reversed by the addition of anisomycin (ANI). The protective effect of JNK-specific inhibitors administered as monotherapy was found to be comparable to that of Met. The aforementioned results suggest that the regulation of the JNK pathway is a critical factor contributing to its protective effect.

**Conclusion:**

The cardioprotective effect of Met in the rat model of MIRI is mediated through the regulation of the JNK pathway and PANoptosis. These findings suggest that Met may provide a potential therapeutic approach for treating MIRI.

## 1. Introduction

Coronary artery disease (CAD) ranks among the leading cardiovascular conditions impacting people worldwide. It has been established as the foremost cause of mortality in both developed and developing nations, causing approximately 17.3 million deaths annually [1]. Acute myocardial infarction is a serious type of coronary heart disease and is a major cause of death and disability worldwide. The preferred treatment for acute myocardial infarction is to use thrombolytic therapy or direct percutaneous coronary intervention (PCI) for timely and effective myocardial reperfusion. However, the reperfusion process itself can cause irreversible and fatal damage to myocardial cells, eventually leading to cell death and increased infarction area, which is called myocardial ischemia–reperfusion injury (MIRI) [2]. Its potential mechanisms may include calcium overload, oxidative stress, mitochondrial dysfunction, apoptosis, and the inflammation-induced death of cardiomyocytes. Intervention with anti-apoptotic and anti-inflammatory drugs may be able to reduce the size of the myocardial infarction area [3]. Clinical practice shows that both remote ischemic preconditioning and postconditioning can reduce the infarct area while its potential mechanism remains to be further clarified [4].

PANoptosis is an inflammatory programmed death pathway named in 2019. It integrates the key characteristics of apoptosis, pyroptosis, and necroptosis but cannot be explained by any one of these pathways alone, forming a regulatory mechanism centered on PANoptosome [5]. The formation and activation of PANoptosome are the key mechanisms of PANoptosis. Including zinc finger protein 1 (ZBP1) PANoptosome, melanoma deficiency factor 2 (AIM2) PANoptosome, mixed lineage kinase domain-like protein (MLKL) PANoptosome, receptor-interacting protein kinase (RIPK) 1 PANoptosome, NOD-like receptor family CARD domain-containing protein 4 (NLRC4) PANoptosome, and NOD-like receptor family pyrin domain-containing 12 (NLRP12) PANoptosome, their structures are like those of inflammasomes. It is composed of specific innate immune sensors, adapters, and catalytic effectors. It has been clearly defined that PANoptosis is involved in the occurrence and development of various diseases, such as infectious diseases, metabolic diseases, immune diseases, neurodegenerative diseases, and tumors, providing new ideas for the treatment of clinical diseases [6].

Metformin (Met), a widely used antidiabetic drug, has recently been found to have significant cardiovascular protective effects [7]. Studies have shown that Met can prevent MIRI by improving mitochondrial function and activating AMP-activated protein kinase (AMPK) to modulate c-Jun N-terminal kinase (JNK)–nuclear factor kappa-B (NF-*κ*B) signaling cascade [8–10]. It has been found that Met exerts anti-inflammation and antiapoptosis effects through AMPK/silent mating type information regulation 2 homolog-1 (SIRT1) signaling pathway and plays a protective role in diabetic cardiomyopathy (DCM) [11]. Recent studies have demonstrated that Met attenuates MIRI through the activation of AMPK, which subsequently inhibits 3-hydroxy-3-methylglutaryl-CoA reductase (HMGCR) expression, thereby reducing the production of reactive oxygen species (ROS) and alleviating oxidative stress and apoptosis in myocardial cells [12]. In another study, the cardioprotective mechanism of Met beyond AMPK activation was elucidated. Research has demonstrated that Met attenuates MIRI-induced ferroptosis through upregulation of nuclear receptor subfamily 4 group A member 1 (NR4A1/Nur77)–mediated isocitrate dehydrogenase 1 (IDH1) and inhibition of the JNK/P38 pathway [13]. Beyond apoptosis and ferroptosis, cardiomyocytes may also exhibit other forms of regulated cell death (RCD), including pyroptosis and necroptosis, with complex interactions occurring among these processes. For instance, activated caspase-1 can cleave caspase-7, and the MLKL can activate NLRP3. Studies have found that during the process of microbial infection, the ZBP1 sensor can simultaneously activate the caspase-8 (apoptosis), gasdermin D (pyroptosis), and MLKL (necroptosis)–related pathways, suggesting that the three RCD patterns may be triggered by a single upstream signal. Therefore, we speculate that PANoptosis may play a more important role in Met alleviating MIRI. The mechanism may involve AMPK activation, NF-*κ*B/JNK signaling pathway activation, ROS generation, etc. However, given the significant role of the JNK signaling pathway in PANoptosis [14, 15], our research primarily focuses on this pathway.

This study is aimed at investigating the anti-PANoptosis properties of Met in rats and exploring whether the activation of JNK plays a role in myocardial PANoptosis. Our results show that Met alleviates MIRI by alleviating the PANoptosis of myocardial cells, and the mechanism is by inhibiting the phosphorylation of JNK and the production of ROS. Our findings may offer new insights into the cardioprotective effects of Met, enhance understanding of its therapeutic potential, and guide the development of new treatment strategies.

## 2. Materials and Methods

### 2.1. Animal

Sprague-Dawley rats weighing 280–320 g were purchased from Beijing Longxue Biotechnology Co. (Beijing, China). All rats were treated and used according to the Guide for the Care and Use of Laboratory Animals (Federal Register File 2011-11490; National Institutes of Health, Bethesda, Maryland). The experimental protocol was approved by the Ethics Committee of Beijing Longxue Biotechnology Co. Ltd (Beijing, China) (Ethics Approval Number: BJLongan-L-L-0002).

### 2.2. Cardiac MIRI Modeling and Met Treatment of Rats

A cardiac MIRI rat model was constructed according to the following procedure [16]: Rats were anesthetized by intraperitoneal injection of pentobarbital sodium (50 mg/kg), followed by tracheal intubation and connection to a rodent ventilator (ALCV9A; Shanghai Alcott Biotechnology Co., Ltd.). Then, the heart was exposed, and a 6-0 silk suture ligated the left anterior descending coronary artery for 30 min. After 30 min ischemic treatment, the slipknot was released to perfuse the heart for 24 h. Ischemia was determined by the blanching of the myocardium and dyskinesia of the chemic region. The sham group rats underwent a thoracotomy but did not receive the MIRI protocol. Met was purchased from Sigma (St. Louis, Missouri, United States) and was dissolved in saline. In order to determine the relatively appropriate dose, combined with relevant literature, intraperitoneal injections of different doses were performed 14 days before the occurrence of MIRI [10]. The rats were divided into three groups: a low-dose group (50 mg/kg body weight), a medium-dose group (100 mg/kg body weight), and a high-dose group (200 mg/kg body weight). The sham and MIRI groups received equal volumes of saline via intraperitoneal injection. For the MIRI + anisomycin (ANI) group, the rats received ANI (a common JNK activator, at a dose of 2 mg/kg) (MilliporeSigma, Germany) after ischemia and reperfusion. For the MIRI + SP600125 group, the rats received SP600125 (JNK inhibitor, at a dose of 20 mg/kg) (Selleck Chemicals, United States) after ischemia and reperfusion.

Then, rats were euthanized to collect the whole heart. The hearts were all stored in liquid nitrogen. The anesthesia and euthanasia process of rats was implemented as follows: First, rats were deeply anesthetized by inhalation of 5% isoflurane. When rats had no response to limb and head stimulation, they were deeply anesthetized. After that, rats were quickly killed by rapid cervical dislocation. After 10 s of rapid cervical dislocation, rats were judged to be dead if they stopped breathing and had no response to limb and head stimulation.

### 2.3. Measurement of Infarct Size

Infarct size was estimated by triphenyl tetrazole chloride (TTC) staining. The hearts were excised and incubated with a 10% KCl solution. After freezing at −20°C, the hearts were sliced and stained with 1% TTC solution (Solarbio Science & Technology Co., Beijing, China) at 37°C for 10–15 min in the dark. The images of these slices were obtained using a digital camera. The infarct area and total area were analyzed by Image-Pro Plus Software 6.0 (Media Cybernetics, Inc., Rockville, Maryland, United States).

### 2.4. Enzyme-Linked Immunosorbent Assay (ELISA)

After reperfusion, blood samples were collected in a 2-mL Eppendorf tube. The serum was separated by centrifugation at 3000 rpm for 10 min at 4°C. ELISA kits were used to measure cardiac troponin I (c-TnI; Solarbio Science & Technology Co., Beijing, China), creatine kinase MB (CK-MB) isoenzyme (Solarbio Science & Technology Co., Beijing, China), interleukin-6 (IL-6; Solarbio Science & Technology Co., Beijing), tumor necrosis factor-alpha (TNF-*α*; Solarbio Science & Technology Co., Beijing), and lactate dehydrogenase (LDH; Solarbio Science & Technology Co., Beijing) according to the manufacturer's instructions. Absorbance at 450 nm was determined using a microplate reader (BioTek Instruments, Inc., Winooski, Virginia, United States).

### 2.5. Detection of Intracellular ROS Content

A total of 40 *μ*L of dimethyl sulfoxide (DMSO) was used to dissolve the ROS detection reagent and prepare the ROS stock solution. The ROS stock solution was subsequently diluted 500-fold with buffer solution to obtain the reaction mixture for further use. Following cell collection and washing, residual liquid was carefully removed from the tube opening using aspiration. Then, 90 *μ*L of fetal bovine serum (FBS) was added to each sample, followed by pipetting to ensure thorough resuspension of the cell pellet. The resulting cell suspension was transferred to a 96-well plate for analysis. Subsequently, 100 *μ*L of the reaction mixture was added to each well, and the plate was incubated in a 5% CO_2_, 37°C cell culture incubator for 1 h. Finally, the 96-well plate was transferred to a fluorescence microplate reader to measure the relative fluorescence intensity at *λ*ex = 490 nm/*λ*em = 525 nm (DCFDA/H2DCFDA - Cellular ROS Assay Kit, Abcam, ab113851).

### 2.6. Western Blot

Total proteins in rat cardiac tissues were extracted by radioimmunoprecipitation assay (RIPA) (Beyotime, Shanghai, China). The lysate was centrifuged, and the concentration of total proteins in each supernatant sample was determined by bicinchoninic acid (BCA) kit (Zeye Biotechnology, Shanghai, China) in line with the directions. The whole protein samples underwent sodium dodecyl sulfate–polyacrylamide gel electrophoresis (SDS-PAGE) for separation. After being transferred onto polyvinylidene fluoride (PVDF) membranes, the proteins were subjected to blockage by 5% skimmed milk for 2 h at room temperature. After that, primary antibody (1:1000) treatment of the proteins was performed for 12 h at 4°C and then secondary antibody (1:2000) treatment for 2 h at room temperature. To assess expression levels of RIPK1 (Abcam, ab300617), p-RIPK1 (Proteintech, No. 66854-1-Ig), RIPK3 (Abcam, ab255705), p-RIPK3 (Abcam, ab222320), MLKL (Abcam, ab184718), p-MLKL (Abcam, ab196436), pro-caspase-1 + p10 + p12 (Abcam, ab179515), NLRP3 (Abcam, ab263899), ASC (Abcam, ab283684), gasdermin D (CST, #39754), BCL2-associated X (Bax) (Abcam, ab32503), Bc1-2 (Proteintech, No. 82469-6-RR), caspase-3 (Abcam, ab32351), ZBP1 (Abcam, ab290736), AIM2 ((Proteintech, No. 20590-1-AP), JNK (Abcam, ab76125), and p-JNK (Abcam, ab239886), phospho-AMPK*α* (Thr172) (CST, #2535), AMPK*α* (CST, #2532), phospho-NF-*κ*B p65 (Ser536) (CST, 3033), NF-*κ*B p65 (CST, 8242), and specific antibodies were employed, respectively.

### 2.7. Immunofluorescence Staining

The histopathological examination method of immunofluorescence staining is as described by predecessors [17]. Paraffin sections were deparaffinized and washed with double-distilled water for 5 min, followed by three times washing with phosphate buffered saline (PBS). The sections were then incubated with 20 *μ*g/mL proteinase K for 30 min and washed three times with PBS. Wheat germ agglutinin (WGA) (GTX01502, GeneTex, United States), TdT-mediated dUTP nick-end labeling (TUNEL) (C1090, Beyotime Biotechnology, Shanghai), gasdermin D, p-RIPK3, and p-MLKL were diluted in 5% BSA at a ratio of 1:200. An adequate amount of the staining solution was then added to completely cover the sections, which were incubated at 37°C in the dark for 1 h. After incubation, the sections were washed three times with PBS. Finally, 30 *μ*L of DAPI solution was added to each section. The slides were then mounted with coverslips and observed under a fluorescence microscope in a dark room to capture images.

### 2.8. Flow Cytometry Analysis

Cardiomyocytes from rats were collected at the same time point and analyzed according to protocols referenced from previous literature [18]. A cell suspension was prepared and transferred to a labeled tube. The suspension was centrifuged at 1000 g for 3 min, and the supernatant was removed. Dulbecco's modified Eagle medium (DMEM) (500 *μ*L/tube) was added to the pellet, and the suspension was gently resuspended. The resulting suspension was then transferred to labeled flow tubes, where Annexin V-PE, 7-AAD-PE-cy7, and caspase-1-FITC antibodies were added. The procedure was strictly followed according to the manufacturer's instructions, and the samples were analyzed on the machine. Each sample was tested with 1 × 10^4^ cells. Flow cytometric data were analyzed using FlowJo software (Becton Dickinson).

### 2.9. Statistical Analysis

All experiments were implemented independently at least three times. Data were processed into means ± standard deviation and analyzed using GraphPad Prism 9.5 software. Two-tailed paired Student's *t*-test performed difference analysis between two groups, and that in at least three groups was carried out by one-way analysis of variance (with Tukey's post hoc test). The threshold for a statistically significant difference was set as *p* < 0.05.

## 3. Results

### 3.1. Met Improved Cardiac Functions of Rats With MIRI

**Figure 1:**
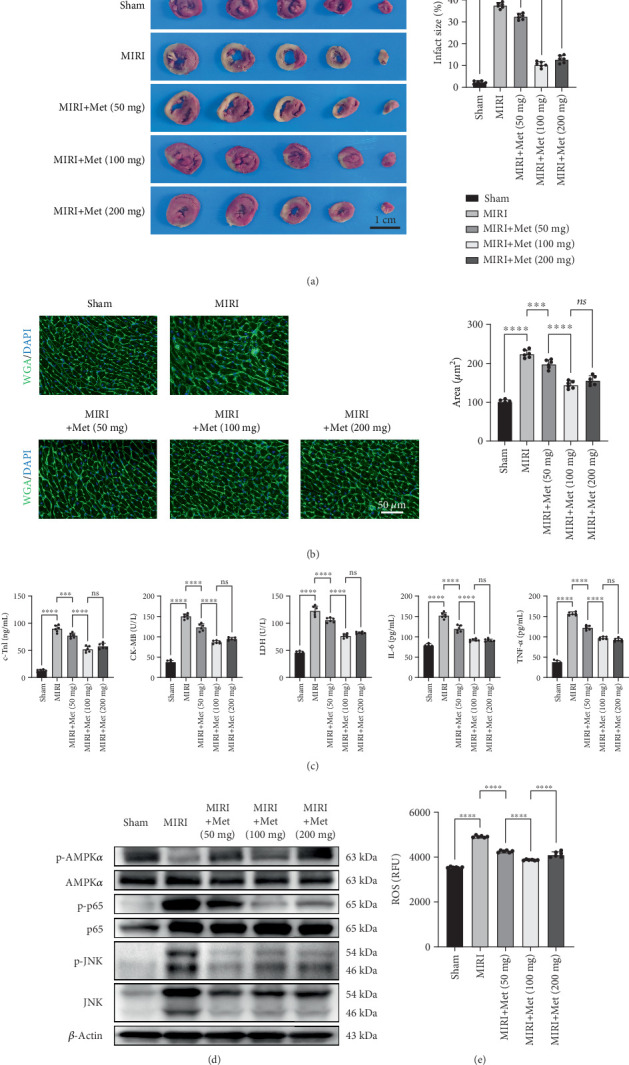
Metformin reduces infarct size and myocardial injury in MIRI rats. (a) Infarct size was evaluated using TTC staining, scale bar = 1 cm, *n* = 6 per group. (b) Myocardial cell hypertrophy was assessed by WGA staining, scale bar = 50* μ*m, *n* = 6 per group. (c) The levels of c-TnI, CK-MB, LDH, IL-6, and TNF-*α* were measured by ELISA, *n* = 6 per group. (d) The relevant signal pathways were detected by Western blot. (e) The intracellular ROS content was measured using a kit, with relative fluorescence intensity detected at *λ*ex = 490 nm and *λ*em = 525 nm, *n* = 6 per group. Statistical significance was determined using one-way ANOVA followed by Tukey's post hoc test. ns, not significant; ⁣^∗^*p* < 0.05; ⁣^∗∗∗∗^*p* < 0.0001. MIRI, myocardial ischemia–reperfusion injury; TTC, triphenyl tetrazole chloride; WGA, wheat germ agglutinin; ELISA, enzyme-linked immunosorbent assay; c-TnI, cardiac troponin I; CK-MB, creatine kinase MB; LDH, lactate dehydrogenase; IL-6, interleukin-6; TNF-*α*, tumor necrosis factor-alpha; ROS, reactive oxygen species.

To evaluate myocardial infarction, we performed TTC staining. The sham group showed normal myocardial tissue with no signs of ischemia or reperfusion injury. In contrast, the MIRI group displayed extensive infarct areas that appeared white, indicating significant infarction due to ischemia–reperfusion. All MIRI + Met groups had significantly smaller infarct areas compared to the MIRI group, suggesting that Met effectively mitigated myocardial injury induced by ischemia–reperfusion. Among the different doses, we found that the medium dose of Met had the most optimal effect, indicating that Met has a protective effect within a certain dosage range (Figure 1a).

Additionally, we used WGA staining to assess myocardial cell morphology. The sham group exhibited intact myocardial cells with clear cell membranes, indicating no damage. The MIRI group showed significant structural disruption and enlarged intercellular spaces, indicating severe injury. The MIRI + Met groups demonstrated better preservation of myocardial cell structure, with tighter intercellular connections, indicating that Met significantly alleviated myocardial damage. The WGA staining results further showed that the medium- and high-dose groups had the best outcomes, with the medium dose showing slightly better results on some indicators (Figure 1b).

To further evaluate myocardial injury, we measured cardiac injury biomarkers including c-TnI, CK-MB, LDH, IL-6, and TNF-*α*. These biomarkers remained within normal ranges in the sham group. In contrast, their levels significantly increased in the MIRI group, indicating severe myocardial injury. Conversely, in the MIRI + Met groups, these biomarkers significantly decreased. This suggests that Met effectively reduced myocardial injury and suppressed the inflammatory response. Among the different doses, the medium dose showed the best overall results, outperforming the high dose on some indicators (Figure 1c). Therefore, we selected the medium dose as the optimal dose for subsequent experiments.

Finally, we assessed the activation of relevant signaling pathways using a Western blot. Studies have demonstrated that AMPK activation in the MIRI group was significantly inhibited compared to the sham group. However, Met, an agonist of AMPK, markedly activated AMPK*α* phosphorylation at Thr172. Additionally, NF-*κ*B activation was higher in the MIRI group than in the sham group, as evidenced by the phosphorylation of p65 at Ser536. Met concurrently inhibited NF-*κ*B activation induced by ischemia–reperfusion. Importantly, JNK was significantly activated during ischemia–reperfusion, and Met treatment reduced JNK phosphorylation induced by ischemia–reperfusion (Figure 1d). The results of intracellular ROS detection indicated that ROS levels in the MIRI group were significantly higher than those in the sham group. Treatment with Met markedly reduced ROS production, with the moderate dose demonstrating the most pronounced effect (Figure 1e). Given the crucial role of the JNK signaling pathway in various forms of RCD and the predominant involvement of this pathway in prior research on Met's mitigation of RCD in cardiomyocytes, our study primarily investigates the activation of the JNK signaling pathway [13, 14].

### 3.2. Met Reduces Myocardial PANoptosis in MIRI Rats by Regulating JNK Phosphorylation

**Figure 2:**
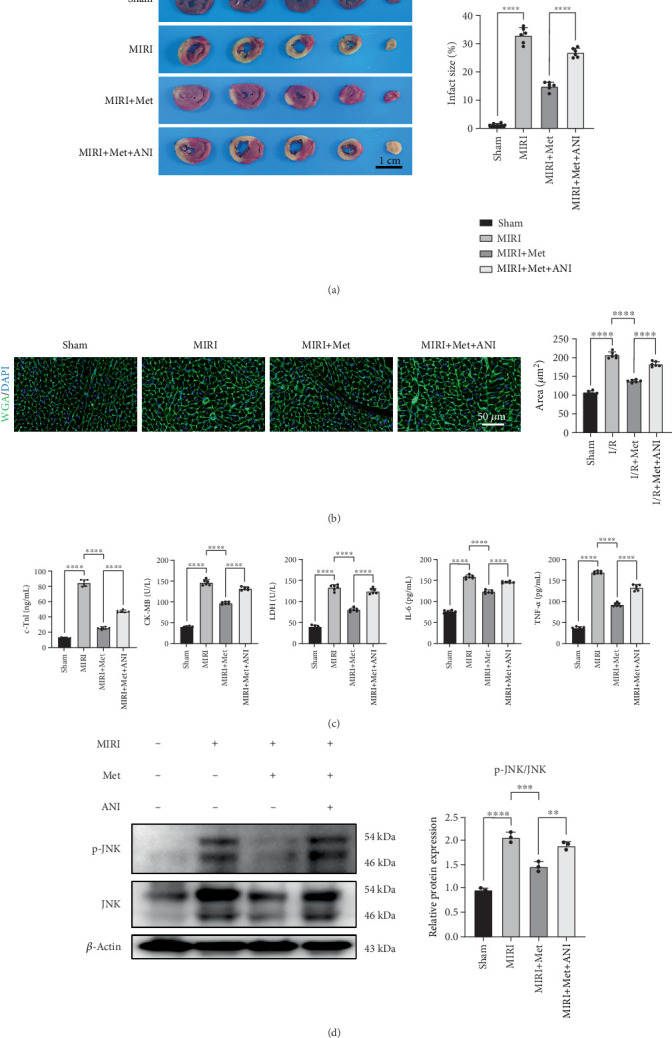
Metformin regulates infarct size and myocardial injury in MIRI rats via inhibiting JNK phosphorylation. (a) Infarct size was evaluated using TTC staining, scale bar = 1 cm, *n* = 6 per group. (b) Myocardial cell hypertrophy was assessed by WGA staining, scale bar = 50* μ*m, *n* = 6 per group. (c) The levels of c-TnI, CK-MB, LDH, IL-6, and TNF-*α* were measured by ELISA, *n* = 6 per group. (d) p-JNK/JNK levels in different groups were analyzed by Western blot, *n* = 3 per group. Statistical significance was determined using one-way ANOVA followed by Tukey's post hoc test. ⁣^∗^*p* < 0.05; ⁣^∗∗∗^*p* < 0.001; ⁣^∗∗∗∗^*p* < 0.0001. MIRI, myocardial ischemia–reperfusion injury; JNK, Jun N-terminal kinase; TTC, triphenyl tetrazole chloride; WGA, wheat germ agglutinin; ELISA, enzyme-linked immunosorbent assay; c-TnI, cardiac troponin I; CK-MB, creatine kinase MB; LDH, lactate dehydrogenase; IL-6, interleukin-6; TNF-*α*, tumor necrosis factor-alpha.

To further evaluate the role of JNK in myocardial infarction, we used TTC staining to assess the effect of the JNK activator ANI. The sham group showed normal myocardial tissue, while the MIRI group exhibited a prominent area of myocardial infarction. In comparison, the MIRI + Met group showed a significant reduction in infarct size, with TTC staining revealing less myocardial damage, indicating that Met effectively alleviated ischemia–reperfusion–induced myocardial injury. The MIRI + Met + ANI group exhibited an increase in infarct size compared to the MIRI + Met group, although it remained smaller than the MIRI group, suggesting that the JNK activator ANI partially reversed the protective effect of Met (Figure 2a).

Similarly, in WGA staining and myocardial injury markers (c-TnI, CK-MB, LDH, IL-6, and TNF-*α*), ANI showed comparable effects. In WGA staining, the MIRI + Met + ANI group exhibited myocardial cell damage between the MIRI group and MIRI + Met group. In terms of myocardial injury markers, the MIRI + Met group showed significantly lower levels of these markers compared to the MIRI group, particularly c-TnI, CK-MB, and LDH, indicating that Met effectively reduced myocardial damage and suppressed inflammation (Figure 2b,c).

We further assessed the expression levels of JNK and p-JNK by Western blot. In the sham group, both JNK and p-JNK expressions were at baseline levels. In the MIRI group, the ratio of p-JNK/JNK was significantly elevated, indicating the activation of the JNK pathway. In contrast, the MIRI + Met group showed a significant reduction in p-JNK/JNK ratio, suggesting that Met inhibited JNK pathway activation. In the MIRI + Met + ANI group, p-JNK/JNK ratio was higher compared to the MIRI + Met group, although it remained lower than in the MIRI group, indicating that the JNK activator ANI partially reversed the protective effects of Met (Figure 2d).

### 3.3. Met Reduces the Occurrence of Myocardial PANoptosis in MIRI Rats

To further evaluate the role of the JNK pathway in MIRI, we utilized Western blot, flow cytometry, and immunofluorescence to deeply analyze the levels of PANoptosis.

**Figure 3:**
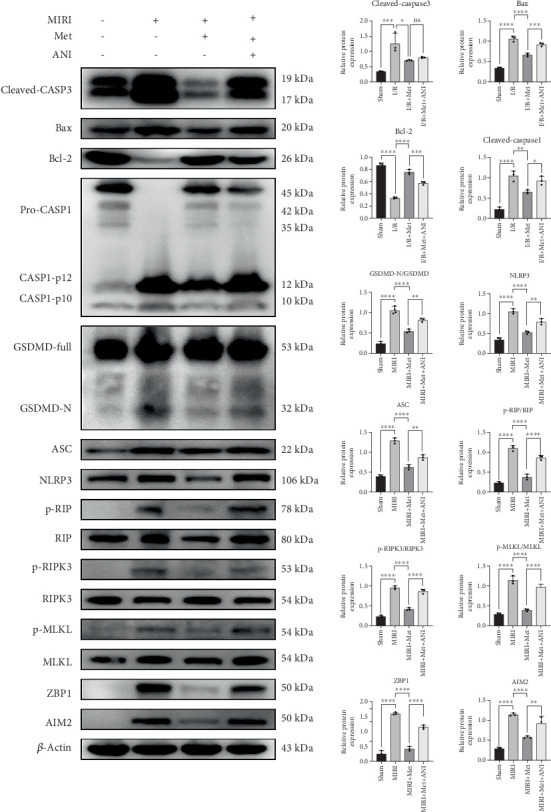
Western blot analysis revealed the expression levels of PANoptotic proteins in MIRI rats, which were regulated by metformin via the JNK signaling pathway. PANoptotic proteins in different groups were analyzed by Western blot, *n* = 3 per group. Statistical significance was determined using one-way ANOVA followed by Tukey's post hoc test. ns, not significant; ⁣^∗^*p* < 0.05; ⁣^∗∗^*p* < 0.01; ⁣^∗∗∗^*p* < 0.001; ⁣^∗∗∗∗^*p* < 0.0001. MIRI, myocardial ischemia–reperfusion injury; JNK, Jun N-terminal kinase; CASP, caspase; Bax, BCL2-associated X; Bcl-2, B-cell lymphoma-2; GSDMD, gasdermin D; ASC, apoptosis-associated speck-like protein; NLRP3, NOD-like receptor pyrin domain containing 3; RIPK, receptor-interacting serine-threonine kinase; MLKL, mixed lineage kinase domain-like; ZBP1, zinc finger protein 1; AIM2, melanoma deficiency factor 2.

In the first place, we assayed the expression of PANoptosis-related proteins, including caspase-1, gasdermin D, ASC, NLRP3, cleaved-caspase-3, Bax, B-cell lymphoma-2 (Bcl-2), p-RIP, RIP, p-RIPK3, RIPK3, p-MLKL, MLKL, ZBP1, and AIM2. In the sham group, the expression of these proteins remained at normal levels. In the MIRI group, the expression of several cell death-related proteins was significantly elevated, particularly cleaved-caspase-1, gasdermin D-N-terminal, ASC, NLRP3, cleaved-caspase-3, Bax, p-RIP, p-RIPK3, p-MLKL, ZBP1, and AIM2. The upregulation of these proteins indicates the activation of PANoptotic mechanisms. In the MIRI + Met group, Met significantly suppressed the expression of these PANoptotic proteins. Notably, Bcl-2 (an antiapoptotic protein) expression increased, while cleaved-caspase-1, gasdermin D-N-terminal, ASC, NLRP3, cleaved-caspase-3, Bax, p-RIPK3, p-MLKL, and p-RIP levels significantly decreased, suggesting that Met mitigated PANoptosis. In the MIRI + Met + ANI group, the JNK activator ANI partially reversed the protective effect of Met, leading to a partial increase in the expression of PANoptotic proteins, although levels remained lower than in the MIRI group. This further supports the notion that the JNK activator ANI partially restored the PANoptotic response induced by myocardial injury (Figure 3a).

**Figure 4:**
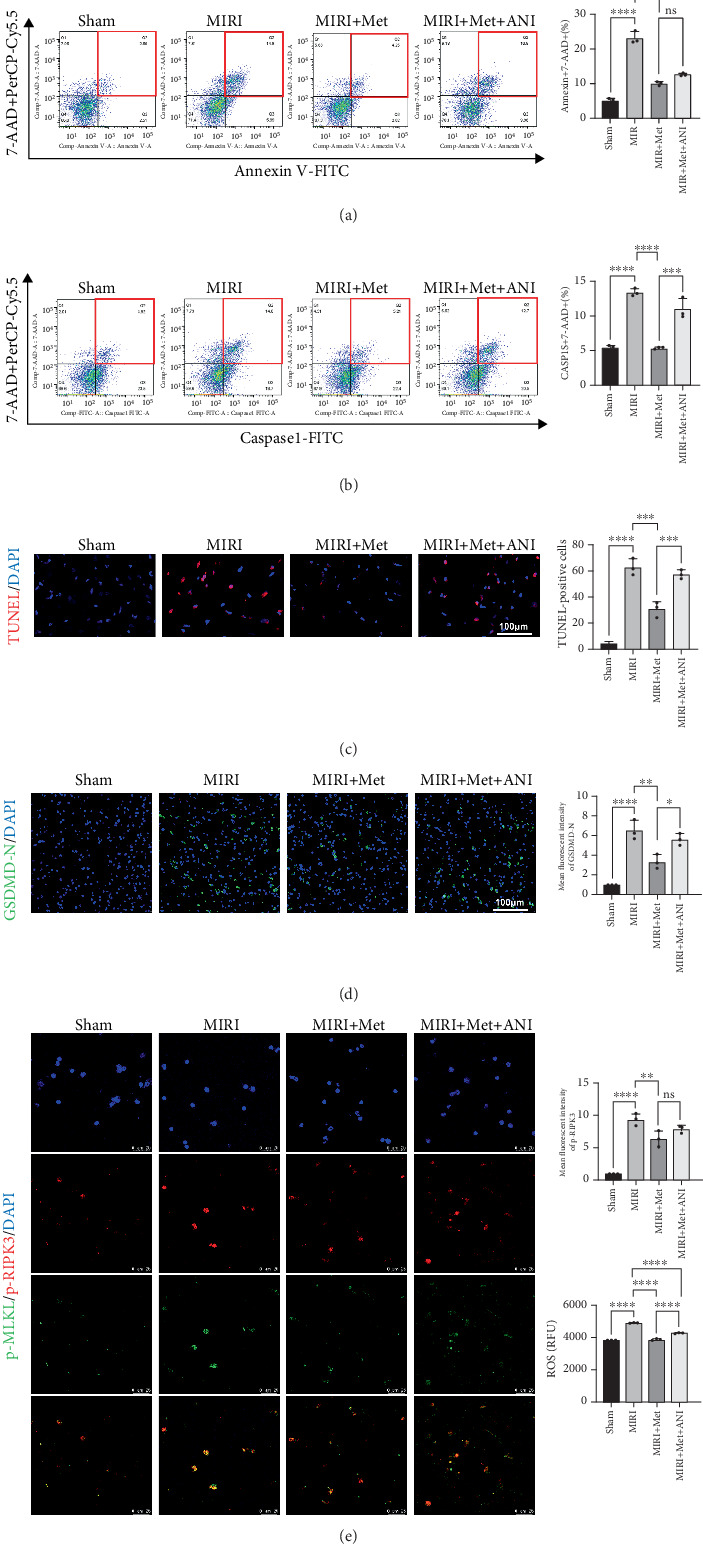
Flow cytometry analysis and immunofluorescence staining were used to study how metformin alleviated the PANopoptosis rate of MIRI rat cardiomyocytes through the JNK signaling pathway. (a) Apoptosis and necroptosis levels of myocardial cells in different groups were detected by flow cytometry, *n* = 3 per group. (b) The levels of pyroptosis in myocardial cells of different groups were detected using flow cytometry, *n* = 3 per group. (c) Apoptosis levels of myocardial cells in different groups were measured by TUNEL assay, scale bar = 100* μ*m, *n* = 3 per group. (d) Gasdermin D levels of myocardial cells in different groups were assessed to detect pyroptosis, scale bar = 100* μ*m, *n* = 3 per group. (e) p-MLKL/p-RIPK3 levels were analyzed to assess necroptosis in myocardial cells in different groups, scale bar = 25* μ*m, *n* = 3 per group. Statistical significance was determined using one-way ANOVA followed by Tukey's post hoc test. ns, not significant; ⁣^∗^*p* < 0.05; ⁣^∗∗^*p* < 0.01; ⁣^∗∗∗^*p* < 0.001; ⁣^∗∗∗∗^*p* < 0.0001. MIRI, myocardial ischemia–reperfusion injury; JNK, Jun N-terminal kinase; TUNEL, TdT-mediated dUTP nick-end labeling; MLKL, mixed lineage kinase domain-like; RIPK, receptor-interacting serine-threonine kinase.

To further evaluate the myocardial PANoptosis rate, we used annexin V and 7-AAD costaining to analyze early apoptosis, late apoptosis, and necrosis, simultaneously stained caspase-1 to detect pyroptosis of the cells. In the sham group, the PANoptosis rate was at the normal level. In the MIRI group, PANoptosis rates significantly increased, suggesting widespread PANoptosis in myocardial cells. In the MIRI + Met group, the PANoptosis rate significantly decreased, indicating that Met effectively reduced PANoptosis. In the MIRI + Met + ANI group, PANoptosis rate increased compared to the MIRI + Met group but remained lower than in the MIRI group, further demonstrating that the JNK activator ANI partially reversed the inhibitory effects of Met on cell PANoptosis (Figure 4a,b).

**Figure 5:**
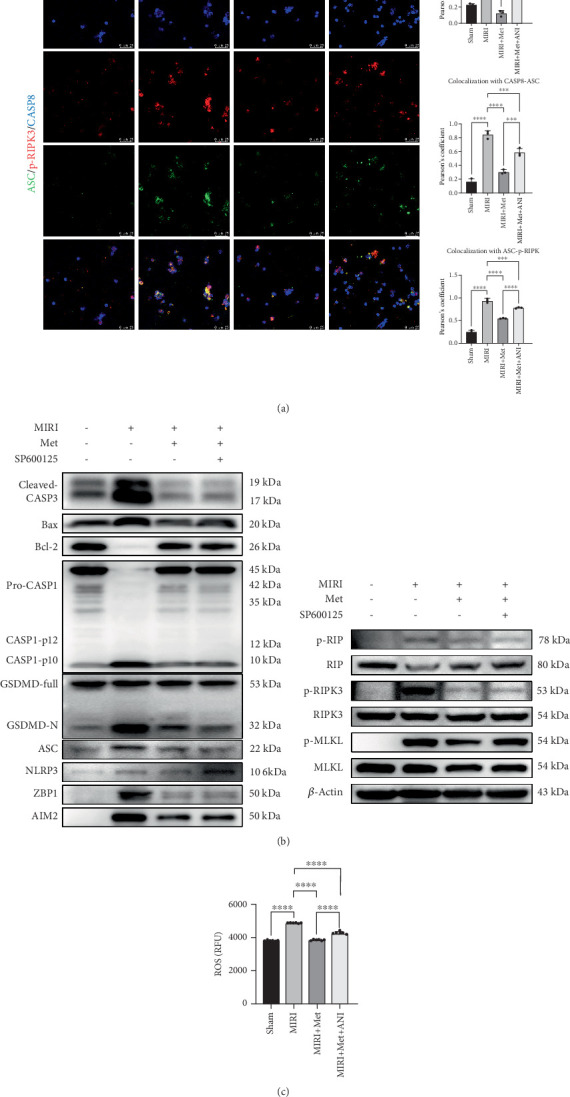
Metformin alleviates cardiomyocyte PANoptosis by inhibiting JNK phosphorylation and reducing intracellular ROS levels. (a) Caspase-8/p-RIPK3/ASC levels were analyzed to assess PANoptosis in myocardial cells in different groups, scale bar = 25* μ*m, *n* = 3 per group. (b) PANoptotic proteins in different groups were analyzed by Western blot, *n* = 3 per group. (c) The intracellular ROS content was measured using a kit, with relative fluorescence intensity detected at *λ*ex = 490 nm and *λ*em = 525 nm, *n* = 6 per group. Statistical significance was determined using one-way ANOVA followed by Tukey's post hoc test. ⁣^∗∗∗^*p* < 0.001; ⁣^∗∗∗∗^*p* < 0.0001. MIRI, myocardial ischemia–reperfusion injury; JNK, Jun N-terminal kinase; ROS, reactive oxygen species.

In the TUNEL, gasdermin D, and p-MLKL/p-RIPK3 double staining immunofluorescence experiments, we observed weak fluorescence signals in the sham group, indicating no significant PANoptosis. In the MIRI group, fluorescence signals for TUNEL, gasdermin D, and p-MLKL/p-RIPK3 were markedly increased, indicating extensive PANoptosis in myocardial cells. In the MIRI + Met group, fluorescence signals for TUNEL, gasdermin D, and p-MLKL/p-RIPK3 significantly weakened, suggesting that Met effectively alleviated myocardial cell PANoptosis. In the MIRI + Met + ANI group, the fluorescence intensity of TUNEL, gasdermin D, and p-MLKL/p-RIPK3 was higher compared to the MIRI + Met group but still lower than the MIRI group, indicating that the JNK activator ANI partially reversed the protective effects of Met (Figures 4c, 4d, and 4e). We further employed immunofluorescence staining to assess the colocalization of caspase-8/p-RIPK3 and ASC. The results demonstrated that colocalization was significantly elevated in the MIRI group compared to the sham group. In contrast, the Met treatment group exhibited reduced colocalization relative to the MIRI group. Notably, the ANI treatment group displayed significantly higher colocalization than the Met group, yet it remained significantly lower than that observed in the MIRI group. These findings suggest that ANI partially attenuates MIRI-induced PANoptosis in cardiomyocytes (Figure 5a).

These findings suggest that Met reduces MIRI by inhibiting the activation of the JNK pathway, which in turn mitigates PANoptotic processes. The JNK activator ANI partially reverses this protective effect, underscoring the role of the JNK pathway in regulating myocardial cell PANoptosis during ischemia–reperfusion injury.

Although ANI is the most used JNK agonist, its extensive biological effects complicate the confirmation of its activation of the JNK pathway. Therefore, we included the JNK-specific inhibitor SP600125 to further elucidate the JNK signaling pathway's role in MIRI-induced PANoptosis of cardiomyocytes. The results indicate that the increased phosphorylation of JNK in the MIRI group may contribute to cardiomyocyte PANoptosis. Met mitigates PANoptosis by reducing JNK phosphorylation. Like the effects observed in the Met treatment group, JNK inhibitors significantly attenuate JNK phosphorylation and consequently reduce cardiomyocyte PANoptosis. These findings further corroborate that the cardioprotective mechanism of Met operates through the inhibition of JNK phosphorylation, thereby suppressing cardiomyocyte PANoptosis (Figure 5b).

Previous studies have demonstrated that mitochondrial dysfunction occurs during MIRI, accompanied by a significant increase in intracellular ROS levels. This phenomenon may be attributed to the suppression of AMPK activation and the concurrent activation of JNK [12, 13, 15]. In this study, a ROS detection kit was employed to quantify intracellular ROS levels under various experimental conditions. The results revealed that ROS levels in the MIRI group were markedly elevated compared to those in the sham group. Notably, Met treatment significantly attenuated MIRI-induced ROS production. Furthermore, when compared with the Met-treated group, the ANI-treated group exhibited a significant increase in ROS levels; however, these levels remained lower than those observed in the MIRI group. These findings suggest that ANI partially counteracts the therapeutic effects of Met (Figure 5c).

## 4. Discussion

**Figure 6:**
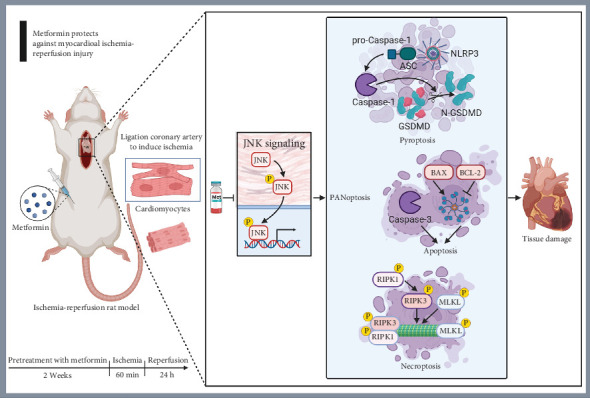
Metformin attenuates myocardial ischemia–reperfusion injury in rats by modulating the JNK pathway and inhibiting PANoptosis mechanisms. Obvious PANoptosis of myocardial cells was observed in the rat model of MIRI. Metformin can effectively alleviate the PANoptosis of myocardial cells and thereby alleviate cardiac injury. The mechanism might be through inhibiting the phosphorylation of JNK. MIRI, myocardial ischemia–reperfusion injury; JNK, Jun N-terminal kinase.

MIRI is characterized by myocardial damage that occurs when coronary blood flow is restored following an ischemic event. This type of injury can lead to cardiomyocyte death and an increase in infarct size [3]. Met is the most used and preferred oral hypoglycemic drug for the treatment of Type 2 diabetes, known for its safety and stable efficacy. Previous studies have demonstrated that Met can regulate multiple signal transduction pathways by activating AMPK and regulate the downstream NF-*κ*B and JNK signaling pathways and can alleviate ischemia/reperfusion injury by regulating energy metabolism, reducing oxidative stress, mitigating inflammatory responses, modulating autophagy, and inhibiting apoptosis [19–21]. Recent research suggests that its cardioprotective effects are related to various forms of RCD in cardiomyocytes, primarily through the inhibition of the JNK signaling pathway [13]. This study indicates that Met mitigates MIRI by reducing PANoptosis in myocardial cells, with the mechanism involving the inhibition of JNK phosphorylation. Our results may provide fresh perspectives on the cardioprotective properties of Met, deepen the understanding of its therapeutic potential, and inform the creation of new treatment approaches (Figure 6).

Met is a widely prescribed drug for the management of Type 2 diabetes and serves as a well-established AMPK agonist. Its primary mechanism of action involves the reduction of glucose levels in the bloodstream. Furthermore, it exerts several beneficial effects on various aspects of cardiovascular health regardless of its hypoglycemic and insulin-sensitizing properties. Met reduces cardiovascular mortality, all-cause mortality, and cardiovascular events in CAD patients through AMPK-dependent or AMPK-independent mechanisms [20, 22–24]. Recent experiments have shown the potential mechanism of Met's cardioprotective effect, including the activation of AMPK, which affects the upregulation of superoxide dismutase (SOD), reduces oxidative stress, and improves mitochondrial function. Furthermore, Met demonstrated antiapoptotic effects, most likely by inhibiting the opening of mitochondrial permeability transition pore (mPTP) and through the anti-inflammatory effect of JNK inhibition [21]. Other studies have also shown that Met can protect sevoflurane-induced neuronal apoptosis by activating the sphingosine 1-phosphate receptor 1 (S1P1)–dependent extracellular signal–regulated kinase (ERK)1/2 signaling pathway [25]. In conclusion, Met mainly protects cardiomyocytes from mitochondrial dysfunction and apoptosis induced by high glucose by activating AMPK and reduces MIRI by reducing inflammation, inhibiting oxidative stress, and improving vascular endothelial function [26, 27]. Our research found that after Met treatment, the myocardial infarction area decreased, the pathological manifestations such as myocardial hypertrophy were alleviated, and at the same time, the levels of serum myocardial injury markers (such as cTnI, CK-MB, and LDH), inflammatory cytokines (such as IL-6 and TNF-*α*), and ROS decreased. These results are basically consistent with the results of predecessors. It indicates that Met not only alleviates cardiac damage but also has a significant anti-inflammatory effect, and this dual effect is crucial in the case of MIRI. In line with previously reported results, our experiment also observed that the Met treatment group exhibited therapeutic effects compared to the MIRI group. However, the therapeutic effect initially increased and then decreased, suggesting the existence of an optimal therapeutic concentration. Therefore, we propose that the lowest effective dosage should be used to minimize side effects while ensuring efficacy [10, 13].

With the discovery of new types of RCD, pyroptosis, necroptosis, and ferroptosis have been proven to be related to MIRI. Recent studies have introduced a unified cell death modality termed PANoptosis, which exhibits molecular features of pyroptosis, apoptosis, and necroptosis but cannot be suppressed by targeting individual pathways alone. The interplay among upper pyroptosis, apoptosis, and necrotosis in MIRI may suggest the involvement of PANoptosis in this pathological process, although further investigation is required for definitive confirmation [28]. Current research provides compelling evidence that significant PANoptosis has been observed in myocardial cells in rat's MIRI models. However, whether Met can also alleviate the newly discovered PANoptosis remains to be further studied. Our results confirm that Met can alleviate MIRI in rats by inhibiting PANoptosis. This discovery is of great significance. Further clarifying its mechanism will provide ideas for the precise prevention and control of MIRI.

Previous studies have found that salvianolic acid B (SAB) inhibits apoptosis by downregulating JNK phosphorylation, the ratio of Bax/Bcl-2, and the expression of caspase-3. Meanwhile, it inhibits ferroptosis in cells by reducing the ubiquitin–proteasome degradation of glutathione peroxidase 4 (GPX4). It indicates that SAB can be used as a myocardial protective agent in MIRI [29]. Another study has found that histone deacetylase HDAC4 silencing can upregulate the expression of miR-206, thereby reducing cardiomyocyte apoptosis, inhibiting oxidative stress, and exerting a protective effect on MIRI through the mitogen-activated protein kinase kinase 1 (MEKK1)/JNK pathway. Inhibiting JNK phosphorylation can reduce cardiomyocyte apoptosis to alleviate MIRI [30]. The above studies indicate that the activation of JNK may play a key role in the RCD of cardiomyocytes induced by MIRI. Our research results confirm that the phosphorylation level of JNK in cardiomyocytes increases after MIRI. Pretreatment with Met can inhibit the phosphorylation level of JNK. Therefore, we speculate that Met can alleviate PANoptosis of cardiomyocytes by inhibiting JNK phosphorylation. To further verify our speculation, we applied the most used JNK agonist, ANI. The results indicated that the JNK activator could partially reverse the therapeutic effect of Met. The therapeutic effect of JNK inhibitors alone is comparable to that of Met, as both can mitigate the widespread apoptosis of myocardial cells. All the above results confirm that in the MIRI model, Met inhibits cardiomyocyte PANoptosis by suppressing JNK phosphorylation.

Previous studies have demonstrated that Met's cardioprotective effects in MIRI are primarily mediated through the activation of AMPK, regulation of the NF-*κ*B/JNK signaling pathway, and modulation of intracellular ROS production. Our findings corroborate this conclusion [12, 13]. The interplay between AMPK activation and the NF-*κ*B and JNK signaling pathways is both intricate and complex [15, 31, 32]. Given the crucial role of the JNK signaling pathway in various forms of RCD and the predominant involvement of this pathway in prior research on Met's mitigation of RCD in cardiomyocytes, our study primarily investigates the activation of the JNK signaling pathway [13, 14]. However, our research also found that JNK agonists can only partially reverse Met's therapeutic effects, indicating that Met may act through multiple pathways. Future research should focus on elucidating the synergistic effects of various intracellular signaling pathways to better understand the mechanism underlying Met's therapeutic effect.

In our research, we observed that inhibiting PANoptosis may offer a novel approach to mitigating the destructive effects of MIRI. Targeting and inhibiting the key components of PANoptosis could reduce cell death and inflammation, thereby improving outcomes in MIRI. Therefore, we need to discuss two pertinent issues. Firstly, can pharmacologic inhibitors of PANoptosis serve as viable alternatives to Met? Theoretically, direct inhibitors can block key signaling pathways of PANoptosis, bypassing the complex metabolic processes mediated by Met, and directly preventing cell death cascades. This approach may offer more targeted and specific strategies for preventing myocardial cell death and reducing MIRI-related inflammation. However, Met is a well-established, pluripotent therapeutic drug with proven safety and efficacy in preclinical experiments and clinical trials. The safety and efficacy of PANoptosis inhibitors remain to be thoroughly investigated. Additionally, PANoptosis is associated with various diseases beyond MIRI, including neurodegenerative diseases and sepsis. Its extensive physiological effects present a challenge for PANoptosis inhibitors to precisely target cardiomyocytes for intervention. Moreover, given the role of PANoptosis in regulating inflammation and immune cell function, long-term inhibition may have unforeseen consequences for the immune response. Therefore, developing safe, effective, and specific PANoptosis inhibitors remains a significant challenge. Next, we need to discuss the potential therapeutic value of combining Met with PANoptosis inhibitors. Given that Met regulates multiple signaling pathways, including those related to PANoptosis, its combination with specific PANoptosis inhibitors may enhance therapeutic efficacy. We believe that this combined approach could be a promising direction for future research and may lead to more effective treatments for MIRI. The combination of Met and PANoptosis inhibitors warrants further investigation. Preclinical studies should focus on evaluating the safety and efficacy of PANoptosis inhibitors in MIRI animal models, both as monotherapy and in combination with Met. Clinical trials should then assess the efficacy of these alternative and combined approaches in patients with MIRI, providing a basis for their potential clinical application.

Despite these encouraging achievements, several questions remain unanswered. Firstly, the exact molecular interaction mechanism among Met, the JNK pathway, and PANoptosis deserves further study. A more detailed understanding of these interactions can pave the way for the development of more targeted treatment strategies. Secondly, our research mainly focuses on the rat model of MIRI, but the translational potential of these findings for human subjects needs to be explored. Future research should be dedicated to confirming these protective effects in clinical settings and evaluating the long-term benefits and safety of Met in patients with MIRI.

In conclusion, this study enhances our comprehension of the cardioprotective mechanisms of Met, highlighting its potential as a therapeutic agent for the management of MIRI. The regulation of the JNK pathway and the suppression of PANoptosis emerge as promising targets for therapeutic intervention, thereby providing optimism for improved clinical outcomes in patients with MIRI.

## 5. Conclusion

This study demonstrates that Met exerts its cardioprotective effects by significantly attenuating MIRI in rats through modulation of the JNK signaling pathway and inhibition of the PANoptosis mechanism. Specifically, Met reduces the secretion levels of myocardial injury markers, inflammatory cytokines, and ROS, while also decreasing the occurrence of PANoptosis in cardiac cells. The JNK agonist ANI partially reverses the therapeutic effects of Met, thereby validating the critical role of the JNK pathway in Met's cardioprotective actions. These findings offer novel insights into the underlying mechanisms by which Met mitigates MIRI and suggest potential therapeutic strategies for the treatment of MIRI.

## Data Availability

The data that support the findings of this study are available on request from the corresponding authors. The data are not publicly available due to privacy or ethical restrictions.

## Nomenclature

CAD: coronary artery disease
PCI: percutaneous coronary intervention
MIRI: myocardial ischemia–reperfusion injury
ZBP1: zinc finger protein
AIM2: melanoma deficiency factor 2
MLKL: mixed lineage kinase domain-like protein
RIPK: receptor-interacting protein kinase
NLRC4: NOD-like receptor family CARD domain-containing protein 4
NLRP12: NOD-like receptor family pyrin domain-containing 12
Met: metformin
AMPK: AMP-activated protein kinase
JNK: Jun N-terminal kinase
NF-*κ*B: nuclear factor kappa-B
SIRT1: silent mating type information regulation 2 homolog-1
DCM: diabetic cardiomyopathy
HMGCR: 3-hydroxy-3-methylglutaryl-CoA reductase
ROS: reactive oxygen species
NR4A1/Nur77: nuclear receptor subfamily 4 group A member 1
IDH1: isocitrate dehydrogenase 1
RCD: regulated cell death
ANI: anisomycin
TTC: triphenyl tetrazole chloride
ELISA: enzyme-linked immunosorbent assay
c-TnI: cardiac troponin I
CK-MB: creatine kinase MB
LDH: lactate dehydrogenase
IL-6: interleukin-6
TNF-*α*: tumor necrosis factor-alpha
DMSO: dimethyl sulfoxide
FBS: fetal bovine serum
RIPA: radioimmunoprecipitation assay
BCA: bicinchoninic acid
SDS-PAGE: sodium dodecyl sulfate–polyacrylamide gel electrophoresis
PVDF: polyvinylidene fluoride
PBS: phosphate buffered saline
WGA: wheat germ agglutinin
TUNEL: TdT-mediated dUTP nick-end labeling
DMEM: Dulbecco's modified Eagle medium
SOD: superoxide dismutase
mPTP: mitochondrial permeability transition pore
S1P1: sphingosine 1-phosphate receptor 1
ERK: extracellular signal–regulated kinase
SAB: salvianolic acid B
Bax: BCL2-associated X
Bcl-2: B-cell lymphoma-2
GPX4: glutathione peroxidase 4
MEKK1: mitogen-activated protein kinase kinase 1

## References

[1] Malakar A. K., Choudhury D., Halder B., Paul P., Uddin A., Chakraborty S. (2019). A Review on Coronary Artery Disease, Its Risk Factors, and Therapeutics. *Journal of Cellular Physiology*.

[2] Hausenloy D. J., Yellon D. M. (2013). Myocardial Ischemia-Reperfusion Injury: A Neglected Therapeutic Target. *Journal of Clinical Investigation*.

[3] Yellon D. M., Hausenloy D. J. (2007). Myocardial Reperfusion Injury. *New England Journal of Medicine*.

[4] Donato M., Evelson P., Gelpi R. J. (2017). Protecting the Heart From Ischemia/Reperfusion Injury: An Update on Remote Ischemic Preconditioning and Postconditioning. *Current Opinion in Cardiology*.

[5] She R., Liu D., Liao J., Wang G., Ge J., Mei Z. (2023). Mitochondrial Dysfunctions Induce PANoptosis and Ferroptosis in Cerebral Ischemia/Reperfusion Injury: From Pathology to Therapeutic Potential. *Frontiers in Cellular Neuroscience*.

[6] Xiong Y. (2023). The Emerging Role of PANoptosis in Cancer Treatment. *Biomedicine & Pharmacotherapy*.

[7] Mohan M., Al-Talabany S., McKinnie A. (2019). A Randomized Controlled Trial of Metformin on Left Ventricular Hypertrophy in Patients With Coronary Artery Disease Without Diabetes: The MET-REMODEL Trial. *European Heart Journal*.

[8] Calvert J. W., Gundewar S., Jha S. (2008). Acute Metformin Therapy Confers Cardioprotection Against Myocardial Infarction *via* AMPK-eNOS-Mediated Signaling. *Diabetes*.

[9] Chen X., Li X., Zhang W. (2018). Activation of AMPK Inhibits Inflammatory Response During Hypoxia and Reoxygenation Through Modulating JNK-Mediated NF-*κ*B Pathway. *Metabolism*.

[10] Palee S., Higgins L., Leech T., Chattipakorn S. C., Chattipakorn N. (2020). Acute Metformin Treatment Provides Cardioprotection *via* Improved Mitochondrial Function in Cardiac Ischemia/Reperfusion Injury. *Biomedicine & Pharmacotherapy*.

[11] Jia W., Bai T., Zeng J. (2021). Combined Administration of Metformin and Atorvastatin Attenuates Diabetic Cardiomyopathy by Inhibiting Inflammation, Apoptosis, and Oxidative Stress in Type 2 Diabetic Mice. *Frontiers in Cell and Development Biology*.

[12] Zhu H., Zhu T., Dubiao D., Zhang X. (2024). Metformin Attenuates Myocardial Ischemia-Reperfusion Injury Through the AMPK-HMGCR-ROS Signaling Axis. *Kardiologiia*.

[13] Wu Z., Bai Y., Chang C., Jiao Y., Chen Q., Guo Z. (2025). Metformin Attenuates Myocardial Ischemia/Reperfusion-Induced Ferroptosis Through the Upregulation of Nur77-Mediated IDH1. *Biochimica et Biophysica Acta (BBA)-Molecular Cell Research*.

[14] Müller K., Honcharova-Biletska H., Koppe C. (2021). JNK Signaling Prevents Biliary Cyst Formation Through a CASPASE-8-Dependent Function of RIPK1 During Aging. *Proceedings of the National Academy of Sciences of the United States of America*.

[15] Shen H. M., Liu Z. G. (2006). JNK Signaling Pathway Is a Key Modulator in Cell Death Mediated by Reactive Oxygen and Nitrogen Species. *Free Radical Biology & Medicine*.

[16] Wang F., Wang H., Liu X. (2021). Neuregulin-1 Alleviate Oxidative Stress and Mitigate Inflammation by Suppressing NOX4 and NLRP3/Caspase-1 in Myocardial Ischaemia-Reperfusion Injury. *Journal of Cellular and Molecular Medicine*.

[17] Zhang X., Zhao D., Feng J. (2021). LuQi Formula Regulates NLRP3 Inflammasome to Relieve Myocardial-Infarction-Induced Cardiac Remodeling in Mice. *Evidence-based Complementary and Alternative Medicine*.

[18] Liu X., Li X., Zhou H. (2023). Changes in Glutamic Oxaloacetic Transaminase 2 During Rat Physiological and Pathological Cardiomyocyte Hypertrophy. *BMC Cardiovascular Disorders*.

[19] Ala M., Ala M. (2021). Metformin for Cardiovascular Protection, Inflammatory Bowel Disease, Osteoporosis, Periodontitis, Polycystic Ovarian Syndrome, Neurodegeneration, Cancer, Inflammation and Senescence: What Is Next?. *ACS Pharmacology & Translational Science*.

[20] Bu Y., Peng M., Tang X. (2022). Protective Effects of Metformin in Various Cardiovascular Diseases: Clinical Evidence and AMPK-Dependent Mechanisms. *Journal of Cellular and Molecular Medicine*.

[21] Higgins L., Palee S., Chattipakorn S. C., Chattipakorn N. (2019). Effects of Metformin on the Heart With Ischaemia-Reperfusion Injury: Evidence of Its Benefits From In Vitro, In Vivo and Clinical Reports. *European Journal of Pharmacology*.

[22] Han Y., Xie H., Liu Y., Gao P., Yang X., Shen Z. (2019). Effect of Metformin on All-Cause and Cardiovascular Mortality in Patients With Coronary Artery Diseases: A Systematic Review and an Updated Meta-Analysis. *Cardiovascular Diabetology*.

[23] McNair B. D., Polson S. M., Shorthill S. K. (2023). Metformin Protects Against Pulmonary Hypertension-Induced Right Ventricular Dysfunction in an Age- and Sex-Specific Manner Independent of Cardiac AMPK. *American Journal of Physiology. Heart and Circulatory Physiology*.

[24] Satyam S. M., Bairy L. K., Shetty P. (2023). Metformin and Dapagliflozin Attenuate Doxorubicin-Induced Acute Cardiotoxicity in Wistar Rats: An Electrocardiographic, Biochemical, and Histopathological Approach. *Cardiovascular Toxicology*.

[25] Yue H., Hu B., Luo Z., Liu M. (2019). Metformin Protects Against Sevoflurane-Induced Neuronal Apoptosis Through the S1P1 and ERK Signaling Pathways. *Experimental and Therapeutic Medicine*.

[26] Huang X. D., Jiang D. S., Feng X., Fang Z. M. (2024). The Benefits of Oral Glucose-Lowering Agents: GLP-1 Receptor Agonists, DPP-4 and SGLT-2 Inhibitors on Myocardial Ischaemia/Reperfusion Injury. *European Journal of Pharmacology*.

[27] Wu Y. (2023). Metformin Inhibits Mitochondrial Dysfunction and Apoptosis in Cardiomyocytes Induced by High Glucose *via* Upregulating AMPK Activity. *Experimental Biology and Medicine (Maywood, N.J.)*.

[28] Xiang Q., Yi X., Zhu X. H., Wei X., Jiang D. S. (2024). Regulated Cell Death in Myocardial Ischemia-Reperfusion Injury. *Trends in Endocrinology and Metabolism*.

[29] Xu X., Mao C., Zhang C., Zhang M., Gong J., Wang X. (2023). Salvianolic Acid B Inhibits Ferroptosis and Apoptosis During Myocardial Ischemia/Reperfusion Injury *via* Decreasing the Ubiquitin-Proteasome Degradation of GPX4 and the ROS-JNK/MAPK Pathways. *Molecules*.

[30] Li Q., Zhu L., Niu F. (2021). Histone Deacetylase HDAC4 Participates in the Pathological Process of Myocardial Ischemia-Reperfusion Injury via MEKK1/JNK Pathway by Binding to miR-206. *Cell Death Discovery*.

[31] Dvoriantchikova G., Ivanov D. (2014). Tumor Necrosis Factor-Alpha Mediates Activation of NF-*κ*B and JNK Signaling Cascades in Retinal Ganglion Cells and Astrocytes in Opposite Ways. *European Journal of Neuroscience*.

[32] Nakano H., Nakajima A., Sakon-Komazawa S., Piao J. H., Xue X., Okumura K. (2006). Reactive Oxygen Species Mediate Crosstalk Between NF-kappaB and JNK. *Cell Death and Differentiation*.

